# Fine-grained Prototype Network for MRI Sequence Classification

**DOI:** 10.2174/0115734056361649250717162910

**Published:** 2025-07-30

**Authors:** Chunbao Yuan, Xibin Jia, Luo Wang, Chuanxu Yang

**Affiliations:** 1 College of Computer Science, Beijing University of Technology, Beijing, China

**Keywords:** MRI sequence classification, Fine-grained learning, Prototype learning, Deep learning, Prototype classification module, Convolutional neural networks

## Abstract

**Introduction:**

Magnetic Resonance Imaging (MRI) is a crucial method for clinical diagnosis. Different abdominal MRI sequences provide tissue and structural information from various perspectives, offering reliable evidence for doctors to make accurate diagnoses. In recent years, with the rapid development of intelligent medical imaging, some studies have begun exploring deep learning methods for MRI sequence recognition. However, due to the significant intra-class variations and subtle inter-class differences in MRI sequences, traditional deep learning algorithms still struggle to effectively handle such types of complex distributed data. In addition, the key features for identifying MRI sequence categories often exist in subtle details, while significant discrepancies can be observed among sequences from individual samples. In contrast, current deep learning based MRI sequence classification methods tend to overlook these fine-grained differences across diverse samples.

**Methods:**

To overcome the above challenges, this paper proposes a fine-grained prototype network, SequencesNet, for MRI sequence classification. A network combining convolutional neural networks (CNNs) with improved vision transformers is constructed for feature extraction, considering both local and global information. Specifically, a Feature Selection Module (FSM) is added to the visual transformer, and fine-grained features for sequence discrimination are selected based on fused attention weights from multiple layers. Then, a Prototype Classification Module (PCM) is proposed to classify MRI sequences based on fine-grained MRI representations.

**Results:**

Comprehensive experiments are conducted on a public abdominal MRI sequence classification dataset and a private dataset. Our proposed SequencesNet achieved the highest accuracy with 96.73% and 95.98% in two sequence classification datasets, respectively, and outperform the comparative prototypes and fine-grained models. The visualization results exhibit that our proposed sequencesNet can better capture fine-grained information.

**Discussion:**

The proposed SequencesNet shows promising performance in MRI sequence classification, excelling in distinguishing subtle inter-class differences and handling large intra-class variability. Specifically, FSM enhances clinical interpretability by focusing on fine-grained features, and PCM improves clustering by optimizing prototype-sample distances. Compared to baselines like 3DResNet18 and TransFG, SequencesNet achieves higher recall and precision, particularly for similar sequences like DCE-LAP and DCE-PVP.

**Conclusion:**

The proposed new MRI sequence classification model, SequencesNet, addresses the problem of subtle inter-class differences and significant intra-class variations existing in medical images. The modular design of SequencesNet can be extended to other medical imaging tasks, including but not limited to multimodal image fusion, lesion detection, and disease staging. Future work can be done to decrease the computational complexity and increase the generalization of the model.

## 1 INTRODUCTION

As a non-invasive medical imaging technology, MRI has received increasing attention in recent years due to its non-invasiveness and good soft tissue contrast. Unlike other imaging technologies such as X-rays and CT, MRI uses magnetic fields and harmless radio waves to produce images, making it a relatively safe imaging method. MRI has good soft tissue contrast and can clearly display the internal structure and tissue information of the human body. MRI is also an important method for diagnosing diseases such as tumors in the liver, heart, and brain [1, 2]. MRI imaging is helpful for the early detection of high-risk groups for tumor diseases in a noninvasive manner, allowing for the determination of appropriate treatment as early as possible. In recent years, deep-learning-based methods provide a further solution for discriminant feature extraction of MRI images.

During an MRI examination, multi-parametric MRI sequences or phrases are typically acquired, each providing diverse information from different perspectives for disease diagnosis. For simplicity, we use 'sequences' to represent both sequences and phases in the remainder of this paper. Accurately identifying MRI sequences is critical for ensuring reliable MRI reporting, especially in MRI intelligent diagnosis assistant systems. For example, it can be used to do sequence distribution or provide prior knowledge for guiding the training of a multi-model diagnosis algorithm. Additionally, it facilitates the generation of highly reliable sequence labels, particularly in dynamic contrast-enhanced (DCE) sequences, where labeling errors may occur due to irregular operations or imprecise timing. To illustrate differences in imaging features of different MRI sequences, this study shows multiple MRI sequence images of multiple patients. As shown in Fig. (**1**), each row of images displays multi-modal MRI sequence images from the same patient. From the first row of images, it can be observed that T1 and T2 sequences exhibit significant differences in tissue signal intensity, indicating that these two sequences have good feature separability. However, in dynamic contrast-enhanced sequences, the DCE-EAP and DCE-LAP sequences of the same patient show high similarity in signal intensity distribution and morphological features. In contrast, the DCE-EAP sequences of different patients exhibit noticeable morphological differences due to variations in individual anatomical structures. This complex distribution characteristic of dynamic contrast-enhanced sequences, characterized by high intra-class similarity and high inter-class variability, poses significant challenges for traditional deep learning methods in feature extraction and classification tasks.

With the development of artificial intelligence technology, deep learning has shown powerful capabilities in the field of medical imaging. By fusing multiple MRI sequences (such as T1-weighted, T2-weighted, and diffusion-weighted imaging), researchers have significantly improved the accuracy of liver segmentation and lesion detection. Deep learning is also widely used in tasks such as lesion detection, organ segmentation, and disease classification. For example, in MRI image analysis, researchers use deep learning models to process multimodal data to improve the accuracy and efficiency of diagnosis. The application of these technologies provides important support for the development of artificial intelligence in medical imaging.

To achieve accurate identification of MRI sequences, the DUKE Liver Dataset [3] was collected and utilized to support exploration of MRI sequence classification using deep learning methods. Currently, researchers [4, 5] have utilized a convolutional neural network (CNN) to classify MRI sequences. However, the MRI sequence classification task has some challenges that are not considered by the mainstream convolutional neural network [6]. These challenges include dealing with large intra-class differences, small inter-class differences, and fine-grained differences among the sequences. First, the classification of different MRI sequences is primarily characterized by fine-grained differences. In MRI sequences classification, different sequences of dynamic enhancement show high similarity. As shown in Fig. (**1**), taking the dynamic contrast-enhanced (DCE) sequences as an example, the main difference between the early arterial phase (DCE-EAP) and the late arterial phase (DCE-LAP) of the same patient lies in the presence or absence of enhancement in the portal vein, with extremely fine-grained differences between the two sequences. These extremely similar sequences pose difficulties for traditional deep learning methods. Then, MRI sequences exhibit small inter-class differences and large intra-class differences, which makes the clustering of similar MRI image features poor. In different sequences within the same MRI examination, since the patient’s posture characteristics have not changed, the interclass differences between sequences within the same examination are small. In the same sequences, differences between different examinations can be significant due to factors such as patient body morphology, age, and scanning parameters. This characteristic of small inter-class differences and large intra-class differences increases the difficulty of deep learning MRI sequence classification.

To address the challenges in MRI sequence classification, we propose a prototype-based classification model named SequencesNet, which leverages fine-grained learning and takes a 3D MRI image as input. The 3D MRI image is composed of multiple MRI slices. The model comprises two primary components: the Feature Selection Module (FSM) and the Prototype Classification Module (PCM). The FSM is designed to identify and extract fine-grained, discriminative imaging features from MRI sequences by filtering out irrelevant representations, thereby enhancing the model's ability to distinguish highly similar sequences and reducing misclassification errors caused by non-pertinent information. Subsequently, the PCM is developed to tackle the complex data distribution characterized by small inter-class differences and large intra-class variations in MRI sequences. It achieves this by taking the fine-grained representations selected by the FSM as input, constructing prototypes, and optimizing classification performance through minimizing the distance between prototypes and samples, thus improving the clustering coherence of MRI sequences. Through the effective combination of FSM and PCM, we can build more clinically interpretable fine-grained features and improve the performance of MRI sequence classification. The contributions of this paper are as follows:

(1) We propose a prototype-based classification model based on fine-grained learning called SequencesNet, designed for accurate MRI sequences classification. Our model can effectively extract fine-grained MRI information and alleviate the problem of poor clustering of MRI sequence categories.

(2) We introduce a Feature Selection Module (FSM) designed to derive detailed and precise features from MRI data through a systematic process of eliminating irrelevant representations. This approach reduces misclassification errors stemming from non-discriminative information. By enhancing the model's capacity to discern subtle distinctions among MRI sequences, the FSM significantly improves classification accuracy, particularly for sequences exhibiting high similarity.

(3) To address the challenge of complex data distributions arising from low inter-class similarity and low intra-class similarity, we developed a Prototype Classification Module (PCM). This module constructs multiple prototype representations and minimizes the distance between these prototypes and their corresponding samples. By doing so, it effectively mitigates the issues caused by small inter-class differences and large intra-class variations in MRI sequences, thereby enhancing the classification accuracy of MRI sequence data.

The remainder of this study is organized as follows. First, section 2 briefly introduces related work. Then, section 3 describes the proposed SequencesNet in detail. After this, section 4 provides and analyzes the experimental results. Next, section 5 offers the conclusions of this study.

## 2 RELATED WORK

In recent years, deep learning has made significant progress in the field of computer vision and image processing, and has been widely used in tasks such as image enhancement, segmentation, and quality assessment. For example, Zhou *et al.* proposed a low-light image enhancement method based on the coefficient of variation (COV), which significantly improved the image enhancement effect by optimizing the Retinex model [7]. In addition, some researchers proposed a reference-free image quality assessment method based on a self-attention mechanism and a recurrent neural network (RNN), which outputs image quality scores by fusing local and global information [8]. Zhou *et al.*, proposed a correlation filter based on adaptive modality weights and cross-modal learning, which improved the robustness of target tracking by fusing visible light and infrared modality data [9]. We first introduce related work in the field of MRI sequence classification. In addition, since our work involves fine-grained image classification and prototype image classification, we also provide a brief introduction to fine-grained image classification and prototype image classification.

### 2.1 MRI Sequences Classification

To the best of our knowledge, research related to MRI sequence recognition is still relatively scarce, with only a limited number of studies documented so far. Due to the varying dimensions of different abdominal MRI sequences, traditional cropping and resizing methods may result in information loss, leading to inaccurate MRI sequence classification. To address this issue, Zhu *et al.* proposed a 3D pyramid pooling network (3DPP Net), consisting of a feature extractor and a 3D pyramid pooling layer [5]. The inclusion of the 3D Pyramid Pooling Layer enables the 3DPP Net to handle MRI sequence images of different sizes and dimensions, effectively mitigating information loss caused by transformations. Additionally, the 3D pyramid pooling layer samples features extracted by the feature extractor in a hierarchical manner, facilitating accurate MRI sequence classification. The model demonstrated favorable performance on the DUKE Liver Dataset [3]. Yang *et al.* [4] achieved MRI sequence classification by leveraging the interaction between multiple MRI sequences obtained from the same examination. The authors initially extracted features from the multiple MRI sequences and performed global average pooling and global maximum pooling operations on these features. The pooled features were then concatenated with the original MRI sequences' features, effectively integrating the features from different MRI sequences and enabling accurate MRI sequence classification. In summary, the field of MRI sequences classification has amassed relevant research accomplishments. However, it has received relatively limited attention and has not garnered sufficient recognition.

### 2.2 Prototype Image Classification

The annotation process for MRI data is time-consuming, resulting in a limited amount of available MRI data for training purposes. Furthermore, the complexity of MRI images introduces challenging sample-specific issues within the MRI data. In order to solve the problem of small samples and hard samples, Snell *et al.* [10] proposed the Prototypical Network, which learns a metric space by computing the Euclidean distance between samples and prototype representations of each class. This approach enables accurate classification even with limited samples. Classification methods based on prototype learning are favored by many researchers because they can achieve ideal classification results with a small number of samples, and have inspired a lot of follow-up work [11-16]. Xi *et al.* [11] introduced a Prototypical Network with cross-scale graph-based semi-supervised learning

to address the issue of overfitting in small-sample scenarios. The author designed a prototype layer consisting of a distance-based cross-entropy loss function and a time-entropy-based regularizer, which can actively enhance the differences and representativeness of node features and prototypes. Zhang *et al.* [17] proposed a global prototype network to solve the image classification problem under limited supervision samples. They transferred the samples from the original data space to the embedded feature space and then used a global representation learning strategy to learn a global prototype representation for each category.

### 2.3 Fine-grained Image Classification

In the classification of natural images, the target objects typically fall into coarse meta-categories, resulting in significant visual dissimilarities among them. In fine-grained image recognition, objects usually come from subcategories of the fine meta-category, with large intra-category differences and small inter-category differences, which makes fine-grained image learning much more difficult than natural image learning [18]. In addition, since there may be sample imbalance between categories, it will further increase the difficulty of the learning tasks. Many different algorithms have been proposed in the research field for fine-grained image learning problems. These algorithms can be mainly divided into localization-based methods and feature encoding-based methods.

Based on localization-based fine-grained image learning methods, key regions within the image are identified and input into the model for learning [19, 20]. In order to obtain the characteristics of key regions in the image, the most direct method is to use segmentation or detection [21-23] to determine the key positions of the image. Zhang *et al.* [24] first used the detection and positioning sub-network to generate candidate regions of the image, and input the candidate regions to R-CNN [23] for classification. This method has achieved remarkable results on multiple fine-grained datasets. Localization-based fine-grained image learning methods achieve fine-grained learning by localizing specific regions within an image. However, this approach heavily relies on the accuracy of the localized sub-network in extracting region-specific information.

Feature encoding-based methods achieve fine-grained feature learning in images through the design of specific loss functions and higher-order interactions [25-28]. Han *et al.* [29] introduced a channel interaction network for fine-grained image classification. Through the incorporation of the channel interaction module, the model can learn interactive connections among channels, and different channels can mutually influence and adapt, enhancing the expressive capability of image representation. However, feature encoding-based algorithms may not fully adjust to new, unseen fine-grained categories. When faced with new categories, the model may struggle to accurately classify them due to the lack of feature encoding information related to the new categories.

## 3 METHODOLOGY

### 3.1 Overview

The framework of SequencesNet is shown in Fig. (**2**). It consists of three main components: Backbone, Improved Vision Transformer, and Prototype Classification Module (PCM). ResNet [30], one of the CNNs with excellent performance, is used as the backbone to extract basic features *F_b_* from the MRI image *I*. We assume that the input image category is q. The Feature Selection Module is added to the Vision Transformer [31] to filter meaningful and important fine-grained features *F_g_* from basic features *F_b_.* The selected fine-grained MRI features *F_g_* are put into the final layer of the vision transformer layer to extract the output CLS. Finally, the Prototype Classification Module (PCM) is used for MRI sequence classification based on CLS.

### 3.2 Improved Vision Transformer

The Improver Vision Transformer primarily consists of two key components: a Vision Transformer and a Feature Selection Module (FSM). The Vision transformer is responsible for extracting features from the *F_b_*, while the Feature selection module refines this process by filtering out fine-grained features *F_g_*. As illustrated in Fig. (**2**), we incorporate the Feature Selection Module into the Vision Transformer architecture. Specifically, the initial N layers of the Transformer are designed to capture long-range dependencies through attention mechanisms. Between the Nth and N+1th layers, we introduce a Feature Selection Module to isolate fine-grained features *F_g_*. These refined features are then fed into the final layer of the Transformer, compelling it to prioritize and focus on detailed, region-specific characteristics.

#### 3.2.1 Vision Transformer

As shown in Fig. (**2**), we use ResNet [30] and Vision Transformer as the main network. Then, ResNet is used to extract the basic features *F_b_* of the MRI image from *I*. The basic MRI features *F_b_* are divided into *M* equal feature tokens. After this, the feature tokens and the corresponding position information are added, and the feature tokens are fed into the linear projection layer to obtain the input 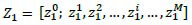 of the first layer of the Vision Transformer. 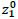 is the input of the CLS token in the first layer of the Vision Transformer Layer. *M* represents the number of tokens. The Vision Transformer encoder consists of *N* +1 layers of multi-head self-attention (*MSA*) and multi-layer perceptron (*MLP*).

Therefore, the input of the *l*-th layer can be represented as Eqs. (**1** and **2**).

**Table:** 

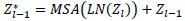	(1)

**Table:** 

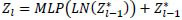	(2)

Where, *LN* represents layer normalization, the attention weights of the multi-head self-attention (*MSA*) in the *l*-th layer can be expressed as Eq. (**3**).

**Table:** 

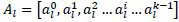	(3)

Where, 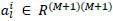, and *k* denotes the number of heads in the multi-head self-attention (*MSA*) of the vision transformer.

#### 3.2.2 Feature Selection Module (FSM)

One of the important challenges in MRI sequences classification is accurately localizing discriminative regions and achieving classification based on subtle differences between different sequences. Inspired by TransFG [32], in order to obtain the fine-grained features

of MRI the Feature Selection Module is proposed to select some important fine-grained features by using the multi-head self-attention of Vision Transformer.

As shown in Fig. (**2**), the Feature Selection Module fuses the attention weights *A_l_* of the first N layers of the Vision Transformer by matrix multiplication. Then, the Feature Selection Module selects fine-grained features *F_g_* as the input of the N+1-th layer of Vision Transformer according to the fused attention, prompting the N+1-th layer of Vision Transformer to focus on fine-grained distinguishable information.

Since the MRI feature *F_b_* is divided into multiple feature tokens, we leverage the attention selection mechanism within the multi-head attention framework to identify feature tokens with high weights, achieving the selection of fine-grained MRI features. The attention weights are derived from the multi-head self-attention mechanism in the Vision Transformer. Specifically, as shown in Eq. (**3**), the l-th layer of the Vision Transformer contains K self-attention heads. As shown in Eq. (**4**), the attention weight of the i-th head can be expressed as 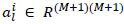.

**Table:** 

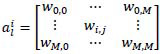	(4)

In 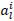, *w*_0,*j*_ represents the attention weight of the CLS token and token *j*. The higher *w*_0,*j*_ indicates that the token *j* and the CLS token have greater attention weight and that token *j* contains more important information. Therefore, we obtain fine-grained features *F_g_* by selecting tokens with high attention weights to CLS tokens.

However, filtering fine-grained features solely based on the attention weights of the Vision Transformer layer's multi-head self-attention is subject to certain constraints and limitations. Prior work by some researchers [33] has provided experimental evidence showing that as the depth of the vision transformer increases, the Vision Transformer continues to weight and fuse different tokens. The attention of the high-level Vision Transformer layer can no longer represent the attention of the CLS token and token *j*. Fine-grained features cannot be selected based on *w*_0_*_,j_*. As shown in the Feature Selection Module in Fig. (**2**), to effectively acquire fine-grained features, we employ a cumulative matrix multiplication technique on the attention weights 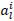 of the preceding *N* Vision Transformer layers. This approach takes into account the interdependencies among different layers and adjusts the distribution of attention weights so that it more accurately reflects the importance of CLS token and other tokens. The specific formula is as follows:

**Table:** 

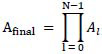	(5)

As shown in Eq. (**6**), 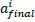 represents the fused attention weights of the *i*-th head after the matrix multiplication of the 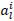. It is important to note that each layer in the vision transformer has K heads. We obtain the attention weights *A_final_* by concatenating the K fused attention weights 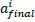.

**Table:** 

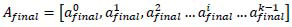	(6)

It is important to note that the size of the fused attention weights 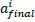 is the same as that of 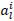 in Eq. (**4**). Through this method of fusing attention weights, we consider the dependencies between different layers to fuse the attention weights and filter fine-grained features based on the fused attention weights.

The CLS token captures the global information of the image and is used as the final output of Vision Transformer. In 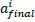, *w*_0_*_,j_* represents the attention weight of the CLS token and token *j*. The higher *w*_0_*_,j_* indicates that token *j* contains more important information. Therefore, we obtain fine-grained features in MRI sequences by selecting tokens with high attention weights to the CLS token according to 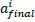. In fused attention weights 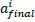, the first column represents the attention weights of all tokens and the CLS token in the *i*-th head attention weights. We select the token corresponding to the maximum weight in the first column of 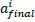 (the red part in Fig. (**2**) as the input of the last Vision Transformer layer. The selected token has more accurate fine-grained information and less redundant information. Since *A_final_* is obtained by matrix multiplication of the attention weights of K heads of the first *N* layers of the Vision Transformer layer, we can obtain K filtered tokens, represented as *F_g_*, as shown in Eq. (**7**).

**Table:** 

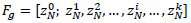	(7)

As shown in Eq. (**7**), 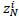 represents the token *i* corresponding to the maximum attention weight in the first column of *i*-th head 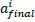.

In addition, the FSM selects fine-grained MRI features. *F_g_* based on the fused attention weights, forcing the last layer of the vision transformer to focus on fine-grained differences in the image and reducing the misclassification caused by irrelevant features.

#### 3.2.3 Prototype Classification Module (PCM)

To deal with the issue of inadequate clustering property of MRI sequences, we proposed the Prototype Classification Module (PCM), which aims to enhance the clustering property. The Prototype Classification Module constructs multiple prototype pools to classify by measuring the distance to the prototypes. This is cial for distinguishing MRI sequences, which have obvious diversity within categories but trivialdifferences between categories. In detail, we constructed a prototype pool, where each sequence category contains multiple prototypes, and classified MRI based on the prototype pools.

The prototype classification module consists of two stages: the prototype pool construction and the prototype-based classification. First, representative central sample features are selected from the training set as the prototype pool for each category. Then, in the prototype-based classification, we compute the distance between the test sample and the multiple prototype pools. The category label of the prototype that is closest to the test sample is determined as the category label of the test sample.

##### 3.2.3.1 Prototype Pool Construction

**Fig. (2):**
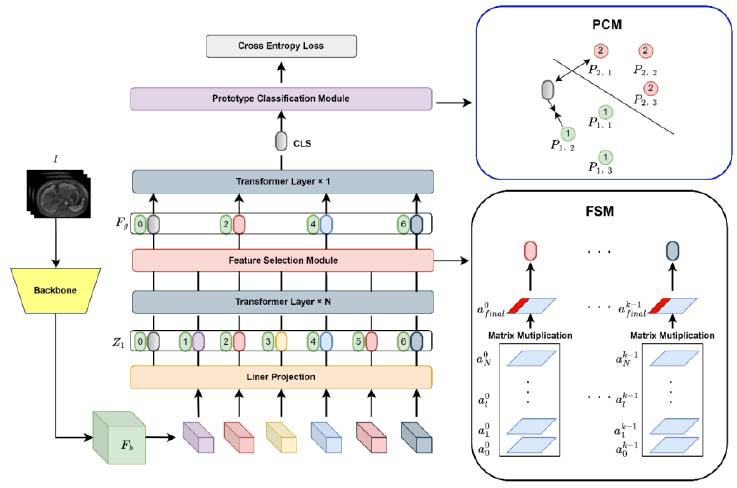
The proposed SequencesNet network. The input to the sequences is a MRI image *I*. basic feature *F_b_* is extracted through a ResNet network, and then *F_b_* is input into impoved vision transformer. After this, fine-grained feature is obtained through Feature Selection Module (FSM). Finally, the Prototype Classification Module (PCM) uses the prototype method to implement MRI sequences classification.

As shown in Fig. (**2**), we replace the prototype classification module with the FC layer and the softmax layer, and then pre-train the model on the sequence classification dataset. Then, the *CLS* of each sample from the pre-trained model is extracted in the training set, and the feature center is calculated for each sequence category. As shown in Eq. (**8**), for sequence category *i*, we get *G* MRI samples features *p_i_* as the prototypes pool with the closest cosine distance to the feature center. The prototypes are denoted as *p_i,j_*, where *i* ϵ [1,2,3*...C*], *C* represents the number of MRI sequence categories, *j* ϵ [1,2,3*...G*], and *G* is the number of prototypes of each sequence, as shown in the top-right corner of (Fig. **2**). We set *G* as 2 in this study. As shown in Formula 9, the multiple prototype pools *R* consist of the prototype set *P_i_* of each category.

**Table:** 

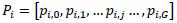	(8)

**Table:** 

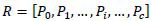	(9)

##### 3.2.3.2 Prototype-Based Classification

As shown in the top-right corner of (Fig. **2**), features from the input MRI sequences are extracted and given as input into the vision transformer to select fine-grained features *F_g_*. Then *CLS* is used to calculate the distance between multiple prototype pools to perform MRI sequence classification. To be specific, the distance between the feature and the prototype features *P_i,j_* is calculated using Eq. (**10**). The sequence category for the MRI image, which is obtained through Eq. (**11**), is the prototype category with the closest distance *DIS_i_* .

**Table:** 

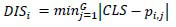	(10)

**Table:** 

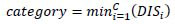	(11)

Herein, we employ a prototype-based classification approach to classify MRI sequences. Prototypes are stored for each sequence category, which are the used for classification. The prototype method can better represent sequence category features and delineate category boundaries. Therefore, the challenges caused by data scarcity and hard samples are effectively addressed. Furthermore, the Prototype Classification Module can fully utilize the fine-grained features extracted by the Feature Selection Module and reduce the chances of misclassification caused by irrelevant features.

#### 3.2.4 Loss Function

The loss function of the proposed method is defined in Eq. (**12**).

**Table:** 

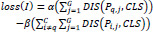	(12)

*α* and *β* are predefined hyperparameters. As shown in Formula 12, the first loss item makes the sequence features of the same category closer in the feature space. For the image of class *q*, the distance between feature CLS of the input MRI image *I* and the sequence prototype *P_q,j_* is minimized Additionally, the second loss item maximizes the distance between CLS and *P_q,j_*, which can push features of different categories farther apart in the feature space. We can minimize the distance between samples and their respective prototypes, ensuring that features belonging to the same class are closer to each other in the feature space, while features from different classes are farther apart, ensuring the separability of MRI samples. In addition, a cross entropy loss function is added to the category classification.

## 4 EXPERIMENTS

### 4.1 Datasets

**Fig. (3):**
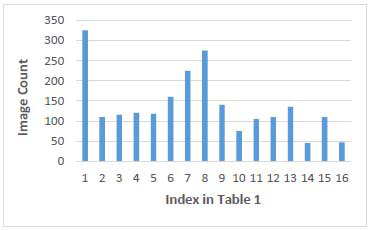
Distribution of the number of MRI sequences. The abscissa represents the sequence's index, which corresponds to Table **1**.

Experiments were carried out in this study to explore the effectiveness of our approach with the DUKE Liver Dataset [3]. The DUKE Liver Dataset comprises typical images from abdominal clinical MRI scans annotated by multiple expert radiologists. It consists of 2,146 sequences from 105 patients, with a total of 16 sequence categories. The distribution of sequences is illustrated in the following (Fig. **3**). The abscissa numbers in the figure correspond to the MRI sequence names in Table **1**. In addition, we constructed 3869 sequences from 384 patients, which were captured from one cooperated Beijing tertiary hospital. Total 10 unenhanced sequences and enhanced phases were included. Manual sequence classification, performed by two radiologists (20 and 10 years of experience) in a consensus reading, was used as the reference standard.

### 4.2 Implementation Details

We randomly divide the dataset into a training set and a test set in a 4:1 ratio. For data augmentation, we applied various transformations to the input images. Specifically, with a probability of 75%, scaling of the width and height of the original images was performed between 0.8 and 1.25. Next, we randomly cropped the MRI images to a size of 48 x 256 x 256 and applied random flipping with a probability of 50%. We utilized the 3D version of ResNet18 [30] as the backbone of SequencesNet and froze its parameters. In the training phase, the number of epochs is set as 300 and the batch size as 48. Then the AdamW optimizer was used with a learning rate of 0.01 and cosine decay as the learning rate update strategy, including a warm-up phase. The number of warm-up epochs was set to 50. Following this, the cosine distance was utilized as the metric (Di) for measuring the distance between the samples and the prototypes. The values of *α* and *β* in Eq. (**12**) were set to 1 and 0.05, respectively. Our model was implemented using PyTorch and trained on 8 A10 GPUs. Due to the limited number of samples, in order to enhance the reliability of the experimental results, all experimental results were averaged by 5-fold cross-validation.

### 4.3 Comparison Results

To evaluate the effectiveness of our proposed model, the comparative experiments were conducted with several fine-grained, prototype, and baseline methods. Among the fine-grained methods, TranFg [32] was chosen as our comparison method, and among the prototype methods, TesNet [34], PIPNet [35] and STProtoPNet [36] were selected. PIPNet can be interpreted as a sparse score table where the presence of prototype parts in the image provides evidence for a certain class. STProtoPNet achieved classification by learning support prototypes that were located close to the classification boundary in the feature space. Three commonly-used metrics were adopted, *i.e.* accuracy, recall, and precision. Finally, the vision transformer [31] and 3D ResNet [30] were compared as baseline methods. Three commonlyused metrics, *i.e.*, accuracy, recall, and precision were adopted.

The comparison results with the other methods in terms of accuracy rate are shown in Table **2**. It has been found that our proposed SequencesNet achieved the highest accuracy of 96.73% and 95.98% in two sequence classification datasets, respectively. In the Duke Liver Dataset, SequencesNet had 2.45% higher accuracy than the prototype method, TesNet, and 4.41% higher accuracy than ResNet18. In our private dataset, SequencesNet also showed 1.81% higher accuracy than TesNet and 2.07% higher accuracy than ResNet18, respectively. In the comparison of prototype methods, SequencesNet showed 0.46% and 0.92% higher accuracies than PIPNet and STProtoPNet in the DUKE Liver Dataset, and 0.10% and 0.12% higher accuracies with the private dataset used in this study, respectively. In addition, we found that for the MRI sequence dataset, the accuracy of Vision Transformer and TransFg was very low. Additionally, we found that the accuracy rates were very low on the vision transformer and TransFg for the MRI sequences datasets.This may be attributed to the limited amount of MRI data and with the use of only vision transformer, it is hard to focus on local regions within the MRI, which play important roles in sequence classification.

To assess the classification performance from the aspect of sensitivity to the positive samples, we also performed a comparison of the recall and precision on each MRI sequence category with the Duke Liver Dataset. As shown in Table **3**, for some typical sub-sequences such as T1-in, and T1-out, ResNet achieved a recall and precision of 1.0. TesNet and TransFg also achieved high accuracy on these sequences. This is because these MRI sequences are quite different, and it is relatively easy to distinguish them. For dynamic enhancement sequences, methods such as TesNet and ResNet did not achieve excellent results. In these dynamic enhancement sequences, different sequences under the same scan exhibit extremely high similarity, and traditional deep learning-based methods do not accurately classify these sequences. As shown in Table **3**, SequencesNet shows extremely high classification ability for multiple dynamic enhancement sequences, due to the Feature Selection Module and Prototype Classification Module.

In order to analyze the performance of our proposed SequencesNet for fine-grained representation, a qualitative comparison was made by the visualization of the class activation maps of SequencesNet, ResNet, and TesNet. As shown in Fig. (**4**), ResNet and SequencesNet have demonstrated their ability to capture vascular details within DCE-LAP and DCE-DP sequences. In the context of DCE-PVP, SequencesNet demonstrates greater capability of capturing information pertaining to the hepatic portal vein. In contrast, TesNet’s attention appears to be distributed more broadly across all three sequences without a specific focus on vascular or portal vein details. The comparison results show that our proposed sequencesNet can better capture fine-grained information. In addition, different MRI examinations of the same patient are also illustrated. As shown in Fig. (**5**), from the first row to the fourth row, the images of different arterial enhancement sequences under the same image are the early arterial phase, late arterial phase, portal venous phase, and delayed phase. The classification results of different models were compared. The red box in the figure indicates that the model correctly predicts the MRI sequence. It can be seen that our model shows better classification results on these extremely similar sequences. Especially for the portal venous phase, SequencesNet can accurately capture the characteristics of the hepatic vein and accurately predict the sequence category.

### 4.4 Ablation Study

To validate the effectiveness of our proposed feature selection module (FSM) and Prototype Classification Module (PCM) for MRI sequences classification, corresponding ablation experiments were conducted on the Duke Liver Dataset. As shown in Table **4**, we examined the effects of different modules on MRI sequences classification by adding different combinations of two modules on the base model. As shown in Table **4**, the classification accuracy of MRI sequences improved by 1% by adding a feature selection module to the base model, where the base model consisted of ResNet and vision transformer. Furthermore, by further adding the prototype classification module (PCM) to FEM, the accuracy increased from 96.32% to 96.73%. The experimental results demonstrate the effectiveness of the prototype classification module for the MRI sequence classification task.

## 5 DISCUSSION

**Fig. (4):**
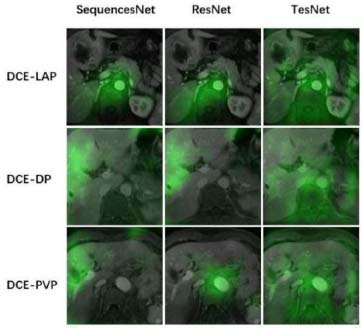
Class activation map visualization.

SequencesNet demonstrates promising performance in MRI sequence classification, excelling in distinguishing subtle inter-class differences and handling large intra-class variability, as validated on the DUKE Liver Dataset and our custom dataset. The Feature Selection Module (FSM) enhances clinical interpretability by focusing on fine-grained features, such as hepatic portal vein details (Fig. **4**), while the Prototype Classification Module (PCM) improves clustering by optimizing prototype-sample distances. Compared to baselines like 3DResNet18 and TransFG, SequencesNet achieves higher recall and precision, particularly for similar sequences like DCE-LAP and DCE-PVP.

However, limitations exist. The FSM’s reliance on Vision Transformer attention weights increases computational complexity, potentially hindering real-time use. At the same time, PCM’s prototype pool quality depends on representative training data, risking bias with imbalanced datasets. Ablation studies confirm FSM and PCM’s complementary roles, with FSM reducing misclassifications and PCM refining category boundaries Table **4**.

Future work could integrate multi-modal data, refine prototype selection dynamically, or extend SequencesNet to joint tasks like lesion detection. While effective optimizing efficiency and data robustness is crucial for clinical translation.

## CONCLUSION

This study proposes a new MRI sequence classification model called SequencesNet, which aims to address the key challenge in medical image analysis: the problem of subtle inter-class differences and significant intra-class variations. SequencesNet uses the attention mechanism to extract clinically meaningful fine-grained features from complex MRI sequences by integrating the feature selection module (FSM) with the Vision Transformer architecture. To further improve the classification performance, the model also introduces a prototype classification module (PCM), which significantly improves the clustering accuracy of features by building a prototype pool and optimizing the distance between samples and prototypes.

Experimental results on the DUKE liver dataset and proprietary datasets show that SequencesNet performs well in MRI sequence recognition tasks, especially when dealing with highly similar sequences. Through systematic ablation studies, the key role of FSM and PCM in improving model performance is further verified.

The modular design of SequencesNet makes it possible to apply it to other medical imaging tasks, including but not limited to multimodal image fusion, lesion detection, and disease staging. However, the model still faces some challenges in practical applications, such as high computational overhead and dependence on high-quality training data. Future research directions will focus on the following areas: optimizing model efficiency to reduce computational costs, exploring multimodal data fusion strategies to enhance the generalization ability of the model, and expanding the application of the model in tasks such as lesion detection and quantitative analysis to further enhance its clinical value. These improvements will enable SequencesNet to play a more significant role in the field of medical image analysis and provide strong support for precision medicine.

## Figures and Tables

**Fig. (1):**
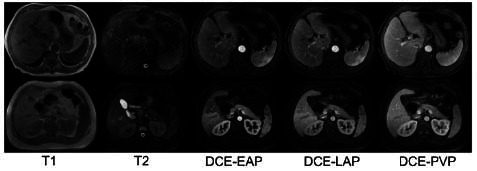
Different MRI sequences images.

**Fig. (5):**
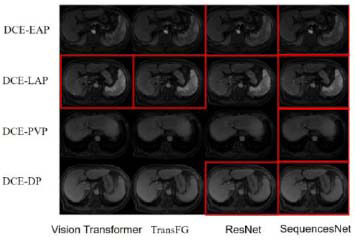
Classification results of SequencesNet.

**Table 1:** Sequence index distribution. Each sequence's name corresponds to the number above it.

Sequences Name			
1	2	3	4
T2	T2-Cor	T1-out	T1-in
5	6	7	8
DWI	MRCP	DCE-PRE	DCE-EAP
9	10	11	12
DCE-EP	DCE-EP-Cor	LOCAL	DCE-PVP
13 OTHER	14 DCE-LAP	15 ADC	16 DCE-TP

**Table 2:** Comparison of SequencesNet and other baseline experiments.

-	Vision Transformer	TransFg	3DResNet18	3DResNet50	TesNet	PIPNet	STProtoPNet	SequencesNet
Duke	76.53%	78.16%	92.32%	90.20%	94.28%	96.27%	95.81%	96.73%
Private	69.30%	73.21%	93.91%	94.17%	94.17%	95.88%	94.78%	95.98%

**Table 3:** Comparison of SequencesNet and other baseline experiments with recall and precision.

**Recall**							
-	ViT	TransFg	3DResNet18	TesNet	PIPNet	STProtoPNet	SequencesNet
T1-in	0.8929	0.8571	0.9643	0.9643	1.0000	1.0000	**1.0000**
T1-out	0.7143	0.7857	0.9643	0.9286	0.9630	0.9626	**0.9643**
DCE-PRE	0.7600	0.6111	0.9815	0.9815	1.0000	1.0000	**1.0000**
DCE-EAP	0.7143	0.7143	0.9286	0.9286	1.0000	1.0000	**1.0000**
DCE-LAP	0.8889	**1.0000**	0.8889	**1.0000**	0.8825	0.8734	0.8889
DCE-PVP	0.3929	0.3929	0.7500	0.6071	0.8547	0.8530	**0.8570**
DCE-EP	0.4839	0.4839	0.8056	0.7419	0.8678	0.8612	**0.8710**
**Precision**							
T1-in	0.7353	0.7500	1.0000	1.0000	1.0000	1.0000	**1.0000**
T1-out	0.9091	0.8462	1.0000	1.0000	1.0000	1.0000	**1.0000**
DCE-PRE	0.8636	0.7674	**0.8833**	**0.8833**	0.8674	0.8625	0.8710
DCE-EAP	0.4167	0.5556	0.8125	0.7222	0.9134	0.9015	**0.9333**
DCE-LAP	0.4706	0.4286	0.5333	0.3333	0.8531	0.8333	**0.8899**
DCE-PVP	0.5000	0.4231	0.8077	0.8095	0.8321	0.8119	**0.8571**
DCE-EP	0.5556	0.5556	0.8929	0.9583	1.0000	1.0000	**1.0000**

**Table 4:** Ablation experiments on the Duke liver dataset. Base represents a network without the FSM and PCM.

-	FSM	PCM	Accuracy
Base			95.31%
Base	√		96.32%
Base	√	√	96.73%

## Data Availability

The data and supportive information is available within the article.

## LIST OF ABBREVIATIONS

MRI: Magnetic Resonance Imaging
DCE: Dynamic contrast-enhanced
CNN: Convolutional neural network
FSM: Feature Selection Module
PCM: Prototype Classification Module
